# Supplementary material for: EnzML: multi-label prediction of enzyme classes using InterPro signatures
Source: BMC Bioinformatics. 2012 Apr 25;13:61. doi: 10.1186/1471-2105-13-61 (PMC3483700; doi:10.1186/1471-2105-13-61)
Supplement: Addtional file 5 — The Java code to format the data files, evaluate and predict. The file enzml_java_code.tar.gz contains the Java code used to format database data to ARFF and XML formats, to execute cross and train-test (jackknife) evaluations and to record evaluation results to database. More information is included in the readme.txt file and the Javadoc files. The code can be used with a MySQL database. To use a different database software, other JDBC drivers might be required. [file 1471-2105-13-61-S5.gz › java_code/utils/doc/uk/ac/ed/inf/utils/guiutils/OptionUtils.html]

OptionUtils


---


|  |  |  |
| --- | --- | --- |
| **Overview** \| **Package** \| **Class** \| **Use** \| **Tree** \| **Deprecated** \| **Index** \| **Help** | |  |
| **PREV CLASS**   **NEXT CLASS** | **FRAMES**    **NO FRAMES**     **All Classes** |  |
| SUMMARY: NESTED \| FIELD \| CONSTR \| METHOD | DETAIL: FIELD \| CONSTR \| METHOD |  |


---


## uk.ac.ed.inf.utils.guiutils Class OptionUtils

```
java.lang.Object
  uk.ac.ed.inf.utils.guiutils.OptionUtils
```

---

``` public class OptionUtils extends java.lang.Object ```

Utilities to manipulate command line options, main args etc.

**Version:**
:   4 Jan 2008

**Author:**
:   Luna De Ferrari luna.deferrari-at-ed.ac.uk

---

**Constructor Summary**

`OptionUtils()`


| **Method Summary** | |
| --- | --- |
| `static boolean` | `getFlag(java.lang.String opt, java.lang.String[] options)` |
| `static java.lang.String` | `getOption(java.lang.String opt, java.lang.String[] options)`             GetOption(String opt, String[] options) gets an option value out of a list of options |

**Methods inherited from class java.lang.Object**

`equals, getClass, hashCode, notify, notifyAll, toString, wait, wait, wait`

**Constructor Detail**

### OptionUtils

```
public OptionUtils()
```


**Method Detail**

### getFlag

```
public static boolean getFlag(java.lang.String opt,
                              java.lang.String[] options)
```

---


### getOption

```
public static java.lang.String getOption(java.lang.String opt,
                                         java.lang.String[] options)
```

:   GetOption(String opt, String[] options) gets an option value out of a
    list of options

    :   **Parameters:**: `opt` - the option that needs to be extracted (e.g. -x): `options` - list of options (e.g. -x 10 -y name) **Returns:**: the value for the option selected (e.g. 10)


---


|  |  |  |
| --- | --- | --- |
| **Overview** \| **Package** \| **Class** \| **Use** \| **Tree** \| **Deprecated** \| **Index** \| **Help** | |  |
| **PREV CLASS**   **NEXT CLASS** | **FRAMES**    **NO FRAMES**     **All Classes** |  |
| SUMMARY: NESTED \| FIELD \| CONSTR \| METHOD | DETAIL: FIELD \| CONSTR \| METHOD |  |


---
